# Notch signalling restores satellite stem cell activity in a model of DMD

**Published:** 2014-08

Duchenne muscular dystrophy (DMD) is an inherited degenerative muscle disease for which there is currently no cure. It is caused by mutations in the gene encoding dystrophin, a cytoskeletal protein involved in maintaining the integrity of sarcolemma (muscle membrane) during muscle contraction. Defects in dystrophin lead to fragility of sarcolemma, which results in muscle dysfunction and degeneration. In normal conditions, satellite cells – a muscle-specific stem cell population – proliferate in response to damage and drive muscle regeneration; however, these cells are progressively depleted in DMD, hampering muscle repair capability. In this study, Shihuan Kuang and colleagues used *mdx* mice (an established model of DMD) to clarify the mechanisms responsible for satellite cell depletion in dystrophy. Whereas in young mice satellite cells could be activated in response to muscle degeneration, their number and self-renewal ability rapidly decreased with age of the mice. The age-dependent decline in satellite cell number and activity was linked with defects in Notch signalling, an evolutionarily conserved pathway involved in muscle stem cell function. Interestingly, artificial activation of Notch signalling resulted in successful restoration of the self-renewal ability of *mdx* satellite cells. This study implicates Notch signalling as a key pathway for preserving stem cell function in dystrophic muscles, and suggests that this pathway might be modulated to improve long-term satellite regenerative function in DMD. **Page 997**

